# Acute kidney injury after contrast-enhanced examination among elderly^1^

**DOI:** 10.1590/0104-1169.3440.2462

**Published:** 2014

**Authors:** Beatriz Bonadio Aoki, Dayana Fram, Mônica Taminato, Ruth Ester Sayad Batista, Angélica Belasco, Dulce Aparecida Barbosa

**Affiliations:** 2 Master's student, Escola Paulista de Enfermagem, Universidade Federal de São Paulo, São Paulo, SP, Brazil; 3 Doctoral student, Escola Paulista de Enfermagem, Universidade Federal de São Paulo, São Paulo, SP, Brazil; 4 PhD, Adjunct Professor, Escola Paulista de Enfermagem, Universidade Federal de São Paulo, São Paulo, SP, Brazil; 5 PhD, Associate Professor, Escola Paulista de Enfermagem, Universidade Federal de São Paulo, São Paulo, SP, Brazil

**Keywords:** Acute Kidney Injury, Contrast Media, Aged, Nursing Care

## Abstract

**OBJECTIVES:**

to assess renal function in elderly patients undergoing contrast-enhanced computed tomography and identify the preventive measures of acute kidney injury in the period before and after the examination.

**METHOD:**

longitudinal cohort study conducted at the Federal University of São Paulo Hospital, from March 2011 to March 2013. All hospitalized elderly, of both sexes, aged 60 years and above, who performed the examination, were included (n=93). We collected sociodemographic data, data related to the examination and to the care provided, and creatinine values prior and post exam.

**RESULTS:**

an alteration in renal function was observed in 51 patients (54%) with a statistically significant increase of creatinine values (p<0.04), and two patients (4.0%) required hemodialysis.

**CONCLUSION:**

There is an urgent need for protocols prior to and post contrast-enhanced examination in the elderly, and other studies to verify the prognosis of this population.

## Introduction

Acute Kidney Injury (AKI) is characterized most commonly by an increase of ≥25% in serum creatinine or an absolute increase of 0.5mg/dL above baseline^(^ 1 ^)^. The main causes of AKI are: ischemia, nephrotoxicity due to antibiotics, heavy metals, organic solvents, poisons, chemicals, anesthetics, endogenous factors, glomerular and vascular diseases, nephritis, diuretics, non-steroidal anti-inflammatory medications and radiographic contrast^(^ 2 ^-^ 3 ^)^. The activity exercised by the latter is the transient increase of renal blood flow, followed by its decrease. It is believed that contrast-induced vasoconstriction is the cause of renal ischemia, a major factor in the pathogenesis of nephrotoxicity^(^ 4 ^)^.

The main signs and symptoms of AKI are: oliguria or anuria, weakness, apathy, loss of appetite, nausea, vomiting, Kussmaul breathing, pulmonary edema, peripheral edema, ascites, cardiac arrhythmias and coma. The treatment of AKI in general involves dialysis and nutritional and fluid therapy^(^ 5 ^-^ 6 ^)^.

The use of contrast media in tests such as angiography, urography or computed tomography can trigger systemic hypersensitivity reactions, cardiac adverse reactions, vascular and renal adverse effects. The amount and nature of contrast used and preexisting risk factors determine the severity of side effects. Contrasts that have high osmolarity are demonstrably more nephrotoxic than those with low osmolarity^(^ 7 ^)^. The main factors of preexisting risk for AKI are: renal failure, diabetes mellitus, heart failure, hypotension, dehydration, and the use of medications such as diuretics and non-steroidal anti-inflammatories^(^ 8 ^)^.

CO_2_ is a type of contrast agent that can be used in exams, such as digital angiography, as it offers a reduced risk of toxicity, and is a safe alternative to iodine-based contrast^(^ 9 ^)^. Barium sulfate, administered orally or rectally, is a contrast agent used in radiological examinations of the esophagus, stomach, intestines, colon and great vessels of the heart, that can trigger toxic reactions in the hours following its administration, such as: nausea, vomiting, diarrhea, abdominal pain, agitation, anxiety, weakness, fainting, sweating, tremors, muscle fibrillation, stiffness of the face and neck muscles, dyspnea, cardiac arrhythmia, paresthesia of upper and lower limbs, convulsions and coma crisis^(^ 10 ^)^.

A study demonstrated that the use of non-ionic contrast-enhanced angiography did not reduce kidney damage in patients who already had impaired renal function after examination, when compared to the use of ionic contrasts^(^ 11 ^)^. A meta-analysis that included twenty-two studies demonstrated a 13.6% incidence of AKI in patients ≥65 years of age^(^ 12 ^)^. AKI is the third leading cause of kidney disease in hospitalized patients, and substantially increases the length of hospital stay, cost of care and intra-hospital mortality^(^ 13 ^-^ 14 ^)^.

Contrast-induced nephropathy (CIN) is an increase of >25% in the baseline levels of serum creatinine. Some studies report that an increase of creatinine occurs within 48 hours^(^ 6 ^,^ 13 ^)^, and others between 48-72 hours^(^ 4 ^,^ 8 ^,^ 15 ^)^ after the administration of the contrast agent.

The various contrast media differ in biochemical and physical aspects, and in regard to their properties related to efficacy. The chemical structure, viscosity, osmolality and ionicity are also considered. The non-ionic compounds and the ones with low osmolarity were considered less nephrotoxic than the hyperosmolar ones^(^ 4 ^,^ 13 ^)^. Yet another type of contrast is being developed, characterized by its nonionic dimers that give the molecule a high affinity with the aqueous medium, thereby decreasing the incidence of nephrotoxicity; such compounds are called isosmolar^(^ 4 ^)^.

Renal lesions caused by the use of contrast are classified into acute tubular necrosis (ATN) and acute tubulointerstitial nephritis (ATIN)^(^ 6 ^)^. The pathophysiology of ATN is multifactor and involves changes in renal hemodynamics and the renal tubules due to a biphasic response: interspersed vasodilation with vasoconstriction which decreases renal blood flow, causing renal ischemia^(^ 8 ^,^ 15 ^)^. CIN is characterized by the damage of epithelial cells of the renal tubules causing decreased renal blood flow that leads to hypoxia in the renal medulla, and also by the appearance of granular cylinders, erythrocytes and inflammatory cells in the urine; ATIN can manifest in a milder form^(^ 6 ^)^.

Examination of renal ultrasound shows that contrast-induced AKI leads to increased renal dimensions. In these cases, scintigraphy with gallium detects inflammatory cells. However, definitive diagnosis is established after renal biopsy^(^ 6 ^)^. For the prevention of contrast-induced AKI, the use of diuretics is indicated, as well as that of vasodilators, hydration, pharmacological vasoconstrictors, and antioxidants inhibitors^(^ 8 ^,^ 16 ^)^.

N-acetylcysteine is the most studied compound in the prevention of contrast-induced AKI. It inhibits the action of free radicals thereby protecting renal function. Prophylaxis is most effective when the medication is administered orally, 24 hours before the procedure^(^ 16 ^)^. A study published in Israel, in 2013, showed no benefit in the use of N-acetylcysteine to prevent contrast-induced AKI, but showed a significant association between the solution volumes administered for the prevention of contrast-induced nephropathy^(^ 17 ^)^.

In elderly subjects, the anatomical and physiological changes in the kidneys, caused by the aging process of the kidney, constitutes an aggravating factor for kidney disease, increasing the susceptibility of renal dysfunction over the years^(^ 4 ^-^ 5 ^)^. A study conducted in São Paulo, with 361 patients, showed that 35% of the elderly presented AKI due to nephrotoxic factors, with one of the predominant causes of nephrotoxicity in this group being the use of contrast for radiologic examinations. The physiology of the kidney in the elderly should be considered when contrast-enhanced exams are requested and performed, since an older individual presents more risks for complications when undergoing invasive procedures and with nephrotoxic medication use. Advanced prognosis of AKI in elderly patients is characterized by oliguria, the need for dialysis, presence of sepsis, and hospitalization in intensive care units^(^ 18 ^)^.

Hydration, use of medications that reduce renal vasoconstriction and oxidative stress, the use of less nephrotoxic contrast, dose adjustment, and discontinuation of medications with nephrotoxic potential should be considered when contrast-enhanced examinations are requested, especially when it comes to the elderly^(^ 19 ^)^.

We understand that knowing the frequency of alterations in the renal function of the elderly after contrast-enhanced examinations can target and support the creation and implementation of protocols for AKI prevention among the elderly population. 

Facing this scenario, the study objectives were: to assess the impact of changes in renal function in elderly inpatients undergoing computed tomography using contrast medium, and, to identify preventive measures of acute kidney injury in the periods before and after the contrast-enhanced CT examination.

## Methods

### Ethical Considerations

The study was approved by the Board of Ethics in Research of the Federal University of São Paulo, under the aforementioned protocol (CEP 1270/09).

### Design, study site and study period

Longitudinal cohort design conducted at the São Paulo Hospital, Federal University of São Paulo -UNIFESP, from March 2011 to March 2013.

### Population

All elderly hospitalized during the study period and who underwent computerized tomography were included in the study. The sample consisted of 93 elderly patients of both sexes, with a minimum age of 60 years.

### Study protocol 

A daily search was made in the CT sector records of all seniors who were examined using contrast media, in addition to the identification of the investigated region in the CT exam, and the type and volume of contrast used. Following this survey, the medical records of these patients were evaluated at the hospital for three days prior to the computed tomography, in order to verify the care provided; and for three days after the CT scan, also to check the care provided and changes in creatinine values. The following data were collected: sociodemographic and morbidity characteristics, indication for examination, exam type, contrast agent and dose used, type of preparation before the examination, care provided after the examination, and serum creatinine values before and after the computed tomography scan.

## Results

Ninety-three elderly who underwent a contrast-enhanced CT scan were studied, with 58 (62.4%) who were male and 35 (37.6%) who were female. The mean age of patients was 70.3 years (60-90 years).

Most of the CT scans were done to examine the thoracic region, followed by abdominal, head, pelvis and other locations. A total of 121 CT examinations were performed, with a mean of 1.3 exams per patient. No standardization was identified in regard to the type of records completed, and the recorded notes varied according to the technician responsible for the examination. Among the 93 records analyzed, 18 (19.4%) did not present any information on the type and dose of contrast used, only in 48.4% of the records were notes found on the volume administered without the contrast agent identified, and in only 30 (32.2%) were data found on the volume and type of contrast used for the examination (Table 1).

**Table 1:** Characteristics of the elderly and of the contrast-enhanced CT scans, São Paulo, SP, Brazil, 2013

Characteristics of the elderly		
Sex		
	Male	58 (62.4)
	Female	35 (37.6)
Age		70.3 (60-90)
CT		
	Thoracic Region	47 (38.8)
	Abdominal	35 (28.9)
	Head	18 (14.8)
	Pelvis	10 (8.3)
	Other	11 (9.1)
Total number of CT scan*		121
Mean number of CT scans per patient		1.3
Records on the use of contrast media		
	No data	18 (19.4)
	Data on the dose used	45 (48.4)
	Data on the dose and type used	30 (32.2)
Types of contrast		
	Ionic	4 (4.2)
	Non-ionic	26 (28.0)

* Some patients had more than one CT Values in numbers (%) and mean value (variation)

During the study, it was found that 33 (35.5%) patients who underwent contrast-enhanced computed tomography did not receive prophylactic measures related to the possible prevention of AKI, while 60 (64.5%) received some type of preparation. Among patients who received some sort of preparation, 46 (49.5%) were intravenously hydrated with normal saline (0.9% NS), 14 patients (15.0%) had 0.9% NS and N-acetylcysteine, and one patient received 0.9% NS followed by increased fluid intake. The mean time for preparation prior to the exam was 26.9 hours.

In the period after the contrast-enhanced exam, 49 (52.7%) patients did not receive any preventive measure, while 44 (47.3%) received some sort of care after the examination. Among patients who received care, 35 (37.63%) were hydrated with 0.9% NS, 7 (7.52%) used 0.9% NS and N-acetylcysteine, and two (3.9%) required hemodialysis after the exam, due to considerable elevation of serum creatinine. The mean time required for care provision after the examination was 57.04 hours. 

Among 24 (25.8%) elderly who underwent CT with contrast, serum creatinine was not measured, and in 69 (74.2%) patients serum creatinine values were found in the records before the exam. The data analysis allowed the observation that the median creatinine value before the examination was 1.00mg/dL, ranging from 0.38 mg/dL to 2.05mg/dL.

The study also showed that the serum creatinine was not requested after the exam for 42 (45.16%) patients. The record control of serum creatinine values before and after the exam was observed in only 51 individuals. The median creatinine after the exam contrast was 1.04mg/dL with changes that ranged from 0.41mg/dL to 6.00mg/dL, featuring considerable elevation of serum creatinine (Table 2). Importantly, two (4%) patients required hemodialysis. The Student's t-test between values of creatinine before and after CT examination enabled a statistically significant finding of p≤0.04, showing that the creatinine values in these patients were above normal values.

**Table 2:** Types of preparations for patients who underwent CT scans, and serum creatinine levels before and after the CT exam, São Paulo, SP, Brazil, 2013

Preparations types and serum creatinin levels		
Preparation before CT		
	No	33 (35.5)
	Yes	60 (64.5)
Hydration with 0.9% NS		46 (49.5)
Hydration with 0.9% NS and N-acetylcysteine		14 (15.0)
Preparation hours		26.9
Care after CT		
	No	49 (52.7)
	Yes	44 (47.3)
0.9% NS after CT		35 (37.6)
0.9% NS and N-acetylcysteine after CT		7 (7.5)
Hemodyalisis after CT		2 (4.0)
Care hours after CT		57.0
Creatinine values before CT		
	No	24 (25.8)
	Yes	69 (74.2)
Median creatinine value before CT		1.0 (0.38-2.05)
Creatinine value after CT		
	No	42 (45.2)
	Yes	51 (54.8)
Median creatinine value after CT		1.04 (0.41-6.0)*

* p value

## Discussion

The increase in life expectancy in Brazil caused a higher incidence and prevalence of diseases such as diabetes, hypertension, cardiovascular disease, stroke and senile dementia, often culminating in hospitalizations and causing an increased need for examinations, including those using contrast media. Males present a greater tendency for developing cerebrovascular disease, acute myocardial infarction and systemic hypertension^(^ 20 ^)^. Aggravating factors that increase this statistic include habits such as: smoking, stress, inactivity, intake of products with high cholesterol, as well as a precarious economic status^(^ 21 ^)^. In the present study we observed concordance with the findings of the abovementioned work, given that 62.4% of the elderly who underwent computerized tomography with contrast were male.

According to the protocol that aims to reduce the rate of contrast-induced nephropathy, patients who undergo contrast examinations should also have serum creatinine level measurement before and two days after the procedure, in addition to the calculation of estimated glomerular filtration rate, use of low-osmolar contrast in case of underlying disease that predisposes renal disorder due to the use of contrast; administration of doses <5 ml/kg/serum creatinine (mg/dl); hydration with 0.9% NS solution of 1 mL/kg/h for 12 hours before and after the examination; oral hydration of ≥2 liters before the exam if the procedure is in outpatient services, and cessation of nephrotoxic medications or medication requiring renal excretion^(^ 19 ^)^.

In the present study it was revealed that the institution where the study was conducted followed no standardized protocol for prevention of renal damage, whereas 35.5% of patients who had CT with contrast were not prepared for the exam, and 52.7% of the sampled population did not receive adequate care after the procedure. There was also no standardization in relation to the exam records.

Records relating to the contrast are extremely important, since several studies that evaluated different types and doses of contrasts demonstrated that their toxicity depended on the degree of osmolarity presented by the agents, i.e., osmolar contrasts are considered less nephrotoxic, as well as non-ionic ones^(^ 19 ^)^. In this study, the number of records that had notes on the type of contrast used in performing the CT examination was 32.2%, and the remaining 67.8% of the analyzed records did not contain records on the type of contrast used. The lack of information may reflect negatively on the decision of possible attitudes related to the prevention of renal damage.

In addition to documenting the type of contrast being of great relevance, studies correlate the amount of contrast administered to the risk of AKI. Other authors also believe that even small dosages could induce renal failure^(^ 22 ^)^. In this study, 19.4% of the analyzed records did not contain records on the volume used, so this refers to the same issue abovementioned, the lack of documentation can interfere with preventive measures to be adopted.

The vast majority of studies on nephrotoxicity related to contrast recommended that all patients should receive oral or intravenous hydration, but few of them compare the use or non-use of this type of treatment. A study that compared three types of preparation, normal saline, normal saline and N-acetylcysteine and sodium bicarbonate, showed no differences between the standard method (saline) and other preparations^(^ 23 ^)^.

Hydration with 0.9% NS for at least 1ml/kg/hour, and if possible 100-150ml/h for 12 hours before and after the procedure, is one of the most appropriate prophylactic measures to prevent alteration of renal function after receiving the contrast. Satisfactory outcomes with the use of normal saline before elective procedures, and even in emergency cases^(^ 19 ^)^, were also obtained. Studies such as this show the importance of hydration prior to contrast studies, however, in this study, only 49.46% of patients received intravenous hydration as has been advocated in the literature.

A study showed that N-acetylcysteine associated with increased hydration was more effective compared to hydration alone. It is known that N-acetylcysteine has a vasodilatory property, so it increases the expression of the nitric oxide synthase enzyme and may prevent contrast-induced nephropathy (CIN) both by reducing the direct oxidative damage and by improving hemodynamics of the kidney^(^ 17 ^)^. 

A study showed that the N-acetylcysteine dosage that should be administered to someone performing contrast-enhanced exam should be 600mg orally 12/12h a day before the exam and 24 hours after the procedure. In emergency cases, one must consider the use of intravenous n-acetylcysteine 150mg/kg in 500ml of normal saline 30 minutes before the procedure and 50mg/kg four hours after it, as recommended by the Rappid trial^(^ 24 ^)^.

Despite advances in studies that show the effectiveness of N-acetylcysteine as a protective measure of the renal function before and after contrast examinations, we did not find the use of this prophylactic measure in most patients' record in the present study. Only 15.05% of patients received N-acetylcysteine before the procedure and 7.52 % used the solution after the exam with contrast media.

One study demonstrated that the combination of medications and hydration provided better renal protection against contrast induced injury than hydration alone, in patients with prior renal damage. Although these authors believed in the beneficial union between N-acetylcysteine and hydration, other conflicting results were found in the literature, which might be explained by the differences among the study population and/or in the diversity of N-acetylcysteine administration protocols. Moreover, one needs to consider findings claiming that N-acetylcysteine may interfere with the tubular transport of creatinine, leading to a decrease in its serum levels^(^ 13 ^)^.

A study compared the concentrations of creatinine and cystatin C, four hours before and 48 hours after oral administration of N-acetylcysteine (600mg, four doses every 12 hours) in 50 volunteers with normal renal function receiving no radiocontrast. There was a significant decrease in mean serum creatinine, while the cystatin C, which is a marker of renal function that is not affected by tubular transport, showed no change^(^ 13 ^)^. 

According to most studies, the lack of preventive measures for the performance of contrast examination provides alteration of renal function that can be of pre-renal origin, due to lack of blood perfusion, since in principle the contrast causes vasodilatation followed by a vasoconstriction. The change in renal function should be diagnosed early because it is reversible, but, if left untreated, it can cause acute tubular necrosis (ATN) and consequently AKI^(5).^

In this study, the control of serum creatinine before and after the contrast examination was observed in only 49.5% of patients, and among the values it was possible to detect a statistically significant increase in creatinine after the use of contrast with a p-value of 0.04. We could not analyze all of the individuals in relation to the dosage of creatinine prior to and post-exam, as some had only pre-scan creatinine values and others only post-scan creatinine measures. Among patients who had the determination of creatinine before and after the examination, only 22.6% received some type of prophylactic measure against impaired renal function.

The fact that an individual has underlying disease is not the only aggravating factor for the occurrence of renal disorders. The elderly are more likely to exhibit risk for contrast-induced nephropathy factors, and among these, patients with chronic renal failure undergoing conservative treatment and patients with diabetes mellitus are serious candidates^(^ 25 ^)^. Other studies have shown that there is an increasing use of contrast media to perform procedures in elderly people, information compatible with the sample of the current study that evaluated individuals >60 years of age. On the other hand, the impact of age as a negative prognostic factor in patients who experience renal disorder is not clear, and there is a tendency in the literature not to consider age as a negative factor in the evolution of these patients^(^ 18 ^)^.

The alterations in the kidney hemodynamics can collaborate, to some degree, with the renal dysfunction, so that some theories are based on the assumption that the aging process of kidney, the higher frequency of pathological conditions in the elderly, the excessive use of medications by elderly patients, and the increased frequency of interventional and surgical procedures are factors that increase the chances of an individual for developing any change in renal function^(^ 18 ^)^. The increase in creatinine values, as claimed by some studies^(^ 6 ^)^, occured within 48 hours; other authors^(^ 13 ^)^ emphasized that this increase may occur 48-72 hours after the patient received the contrast. Our results show that in 25.8 % of hospitalized patients who had a CT scan, the creatinine values was not collected prior to the injection of contrast; in 45.2 % of the participants, the serum creatinine was not checked after the administration of contrast - even with the scientific evidence of risks experienced by patients undergoing contrast examinations.

The limitations of the study highlight that, despite the implemented protocol in the institution on the performance of contrast-enhanced CT scan, neither is this protocol fulfilled nor are there routines for documenting data related to such contrast examinations. 

We considered the conduct of further prospective studies in the institution of fundamental importance, so that it can implement effective protocols for prevention of renal impairment in elderly patients who undergo contrast-enhanced examinations, avoiding the risk of progression to chronicity.

## Conclusions

The current study showed that many elderly had contrast-enhanced examination; however, the protocol on possible preventive measures of renal dysfunction that was implemented in the institution was still not being completed. Despite hospitalization, we did not find clear documentation about the preventive measures that should be used before and after the performance of contrast examinations in this age group. 

The lack of appropriate documentation in some cases in regard to the type and volume of contrast used in these patients was a limitation of the study. There is an urgent need for protocols related to prophylactic measures prior to and post contrast-enhanced examinations in the elderly, as well as more prospective studies to define the prognosis of elderly patients with contrast-induced acute renal disorders.
